# When and how to use predictive biomarkers for corticosteroid treatment of septic shock

**DOI:** 10.1186/s13054-018-2254-z

**Published:** 2018-11-21

**Authors:** James A. Russell

**Affiliations:** 1 Centre for Heart Lung Innovation, St. Paul’s Hospital and University of British Columbia, 1081 Burrard Street, Vancouver, BC V6Z 1Y6 Canada; 2 Division of Critical Care Medicine, Vancouver, BC V6Z 1Y6 Canada

**Keywords:** Septic shock, Predictive biomarkers, Gene expression, Leukocyte

The decision to give glucocorticoids to patients who have septic shock is difficult because of conflicting randomized controlled trial (RCT) level I evidence. Nonetheless, there is some evidence of overuse of corticosteroids in septic shock.

Early cohort studies found that there was an acquired corticosteroid deficiency in septic shock. A subsequent RCT [1] found that corticosteroids lowered mortality in patients who had an abnormal (i.e., inadequate) response to adrenocorticotropic hormone (ACTH) stimulation in septic shock. The ACTH stimulation test has false positives and false negatives and is not recommended for deciding whether or not to administer glucocorticoids in septic shock [2]. Two large multicentre RCTs of corticosteroids in adult septic shock had conflicting results; Ananne and colleagues [3] found significant benefit while Venkatesh and colleagues [4] found no effect of corticosteroids on mortality. The most recent Surviving Sepsis Campaign (published prior to Annane [3] and Venkatesh [4]) used a cautionary tone, recommending *against* corticosteroid use in patients who have responded adequately to norepinephrine [5]. Similarly, but in a more positive tone, the recent Guidelines for the Diagnosis and Management of Critical Illness-related Corticosteroid Insufficiency (CIRCI) [2] recommend *for* corticosteroids in patients who do not respond to norepinephrine in septic shock.

One reason for conflicting evidence regarding responses to corticosteroids in septic shock is that different patients have different genomic, transcriptomic, and proteomic profiles that define different responses to corticosteroids in septic shock. Alder and colleagues recently made the hypothesis that peripheral leukocyte glucocorticoid receptor (GCR) expression and serum cortisol levels correlate with the response to glucocorticoids in pediatric septic shock (REF). They measured these biomarkers in a modest size prospective cohort (*n* = 164) of children who had systemic inflammatory response syndrome (SIRS), sepsis, or septic shock. The GCR expression levels were lower and the serum cortisol levels were higher in patients who had poorer outcomes. Where does this study leave the clinician who cares for patients with septic shock?

Finding predictive biomarkers—i.e. pharmacogenomic, transcriptional, and proteomic biomarkers that identify patients with improved responses to an intervention—is the holy grail of septic shock management [6]. We found that a novel combination of serum cytokine levels predicted improved responses to glucocorticoid administration in adult septic shock [7]. However, our study and others were made using cohorts of non-randomized patients who were treated with glucocorticoids or were simply observational cohorts such as Alder and colleagues (REF). Accordingly, despite the potential uses of predictive biomarkers in septic shock, the Surviving Sepsis Campaign [5] does not recommend any predictive biomarkers.

Transcriptomics—or expression profiling—is the study of RNA transcripts that are produced by the genome in specific conditions at specific times in specific tissues. So transcriptomics is more complex and more dynamic than genomics in that our genome is set at conception while transcriptomics change hour by hour in septic shock. Transcriptomics advocates cite that an advantage of transcriptomics is that they are even more specific than genomics and thus are better candidate predictive biomarkers. However, septic shock transcriptomics studies face barriers for robustness, such as which tissue to sample when—septic shock is a very rapid process (leading to lead time bias in clinical studies)—and even establishing a gold standard for the diagnosis of septic shock [8]. Alder and colleagues used peripheral blood leukocytes—which express the GCR but are a surrogate for deeper tissues of interest—drawn within 24 h of onset of SIRS, sepsis, or septic shock and then measured GCR expression by conventional flow cytometry because they were studying a very limited number of expression transcripts. Several groups [9] have evaluated genomics or whole blood or specific leukocyte gene expression as diagnostic and prognostic biomarkers in septic shock. Transcriptomics has identified subtypes of acute kidney injury (AKI) [10], a common complication of septic shock.

What are the next steps for validation of GCR expression as a predictive biomarker of corticosteroid administration in septic shock? Corticosteroids need to be evaluated in RCT(s) that are adequately powered to detect a significant interaction between (1) GCR expression and (2) use—or not—of corticosteroids. Some would argue, in part because of the storied controversy of steroids in septic shock, for a second confirmatory RCT to validate a GCR expression predictive biomarker. Recently completed RCTs such as those by Annane and colleagues [3] and Venkatish and colleagues [4] are excellent choices for validation because both were rigorous, yet the former was “positive” while the latter was “negative”. I suggest that if a GCR expression biomarker significantly predicted which patients responded positively to glucocorticoids in both RCTs, even steroid skeptics would be interested.

After such confirmation and prior to widespread clinical use, many would recommend regulatory approval of a clinically validated GCR kit.

We and others have similarly addressed predictive biomarkers such as genomics [11], cytokine levels [12], and proteomics for use of vasopressin in septic shock. This is relevant because vasopressin treatment is also controversial in septic shock; the largest RCTs [13, 14] of vasopressin in septic shock were “negative” but there were suggestions of efficacy in patients who had less severe septic shock [13]. Similarly, genomics of the β1 adrenergic receptor could identify good responders to the first line vasopressor in septic shock, norepinephrine [15].

Let’s actualize a future in which individual patient baseline profiling of pharmacogenomics, mRNA expression (e.g., GCR), and protein levels (e.g., cytokine and cortisol levels) could *personalize* treatment with corticosteroids, vasopressin, and norepinephrine in septic shock (Fig. 1) and even guide post-discharge care to decrease the readmission risk. This future is not far off but requires focused design, execution, and analysis of well-conducted predictive biomarker studies in already completed and future RCTs.

**Fig. 1:**
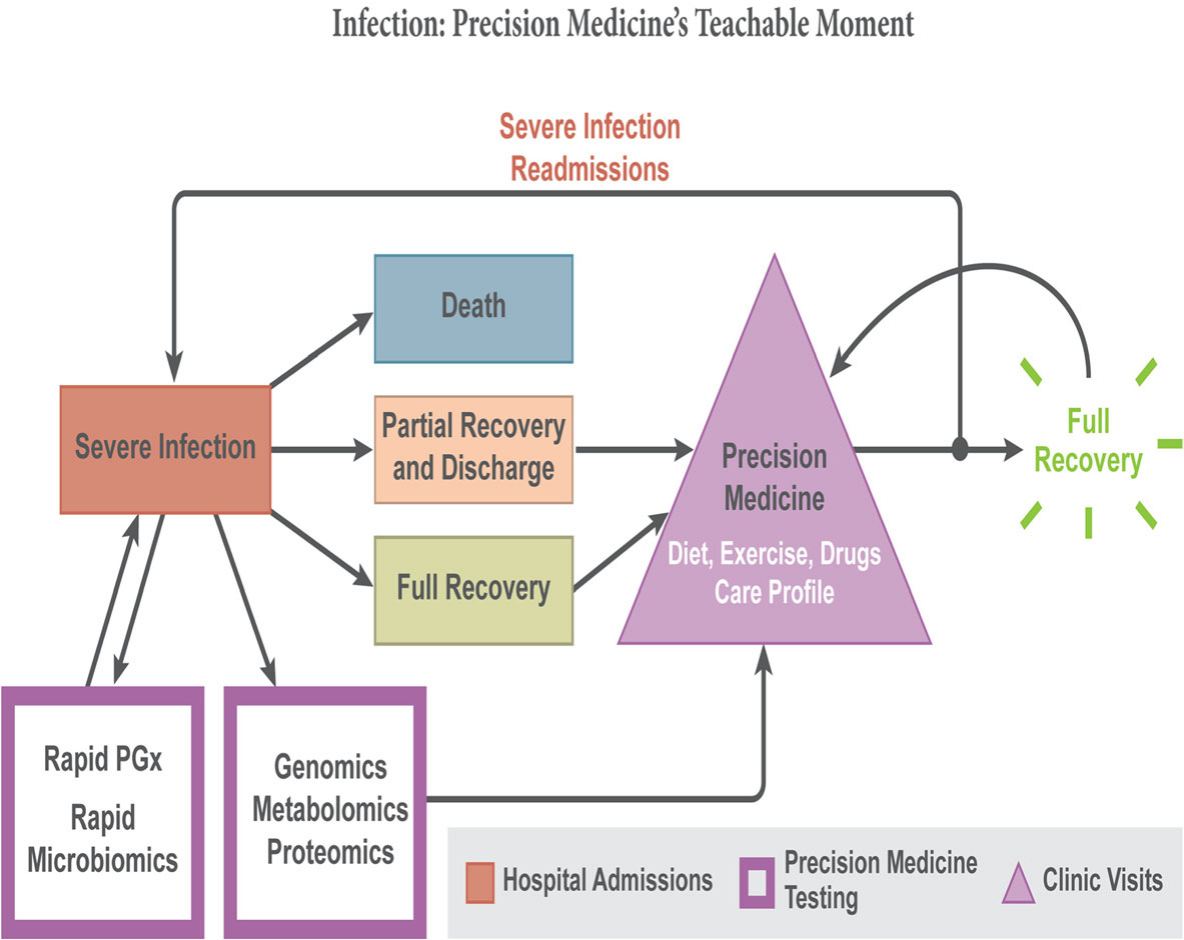
In the future, patients who have severe infection will have rapid pharmacogenomics (*PGx*), microbiomics, genomics, metabolomics, and proteomics (‘omics) at presentation to guide acute management. Patients then die, have a partial or full recovery, and are discharged. After discharge, patients will have follow-up in a precision medicine clinic or office where the results of the ‘omics will be discussed to select a diet, exercise, and drugs profile for each patient. This will enhance the chances for full recovery and reduce the risk of a readmission for severe infection

## Abbreviations

ACTH: Adrenocorticotropic hormone
AKI: Acute kidney Injury
CIRCI: Critical illness-related corticosteroid insufficiency
GCR: Glucocorticoid receptor
RCT: Randomized controlled trial
SIRS: Systemic inflammatory response syndrome
